# Assessing macrofungal diversity to identify conservation priority areas: a case study from the southern section of the Hengduan Mountains, China

**DOI:** 10.3897/imafungus.17.168781

**Published:** 2026-06-12

**Authors:** Shi-Liang Liu, Dong-Mei Liu, Shan Shen, Xue-Wei Wang, Qian-Zhu Li, Jia Yu, Xing-Chun Li, Li-Wei Zhou

**Affiliations:** 1 State Key Laboratory of Microbial Diversity and Innovative Utilization, Institute of Microbiology, Chinese Academy of Sciences, Beijing 100101, China Institute of Microbiology, Chinese Academy of Sciences Beijing China; 2 The Institute of Ecology, Chinese Research Academy of Environmental Sciences, Beijing, China The Institute of Ecology, Chinese Research Academy of Environmental Sciences Beijing China; 3 University of Chinese Academy of Sciences, Beijing 100049, China University of Chinese Academy of Sciences Beijing China

**Keywords:** Community phylogenetics, elevation gradient, funga, priority area, species identification

## Abstract

Biodiversity has provided enormous sustainable advantages for human beings, but is facing extinction threats under the pressure of global environmental changes. Nevertheless, although macrofungi play a crucial role in forest and grass ecosystems, there is no standard procedure for evaluating macrofungal diversity in priority areas for ecological protection, which could support scientific and feasible conservation strategies. Here, we selected the southern section of the Hengduan Mountains as a sampling region and conducted systematic field surveys on macrofungi. From 177 sampling sites, 1771 macrofungal specimens were collected. Through integrated morphological and sequencing (ITS and nLSU regions) analyses, 809 species were accurately identified, belonging to two phyla, nine classes, 28 orders, 123 families, and 336 genera. We found that the high, mid and low elevation groups in the sampling region had no significant difference in macrofungal species richness and phylogenetic diversity, and shared a random process of community assembly mainly shaped by ecological habitats. However, the three elevation groups had lots of unique species, significantly different community structures, and unique indicator species. More importantly, the currently established protected areas may not truly protect the full extent of the macrofungal diversity in this region. Accordingly, we encourage the establishment of more nature reserves specifically dedicated to unique macrofungal species identified in non-protected areas, along with the development of long-term monitoring programs focusing on indicator species to track the spatiotemporal dynamics of macrofungal communities. Beyond the southern section of the Hengduan Mountains, this study proposes an approach for conducting macrofungal diversity assessments that could potentially be used to identify areas for conservation, and which we hope will be taken up by the scientific community and adapted to other regions and types of ecosystems in future work.

## Introduction

Biodiversity is an invaluable natural benefit to human beings (Naeem et al. 2016). Many organisms provide enormous sustainable advantages for humans in various ways, such as yielding food, providing raw materials for textiles and clothing, synthesizing antibiotics and so on, and thus can be treated as strategic biological resources (Bai et al. 2023). Nevertheless, the continuous global environmental changes caused mainly by human activities raise global concerns about biodiversity loss (Dirzo and Raven 2003). It is estimated that about one million species are facing extinction threats (Tollefson 2019), which highlights the urgent need for effective conservation strategies to ensure the sustainability of Earth’s biological heritage (Hu et al. 2021; Wei 2021).

Fungi are the second most speciose group of organisms after insects on Earth, and are distributed almost everywhere (Purvis and Hector 2000; Zhou and May 2023). This successful evolutionary history indicates that fungi play a crucial role in multiple ecosystems. However, the current knowledge of fungal diversity, distribution and conservation is still much poorer than that of plants and other well-studied taxonomic groups (Niskanen et al. 2023). For example, according to the International Union for Conservation of Nature (IUCN) Red List (IUCN 2025), assessments have been completed for 76,864 plant species and 64,754 vertebrate species. In comparison, only 1,302 fungal species have been assessed, meaning that fungi represent just 0.8% of all species currently evaluated on the Red List. By contrast, the percentages for plants and vertebrates are approximately 18% and 84%, respectively. This knowledge gap in Red List assessments, referred to as the Scottian shortfall (Haelewaters et al. 2024), limits the formulation of effective fungal conservation policies (Liu et al. 2024a).

Macrofungi are a group of fungi producing fruiting bodies visible to the naked eye (Mueller et al. 2006; Wang et al. 2021). As an essential component of forest and grass ecosystems, macrofungi play a crucial role in facilitating energy flow and nutrient cycling via interaction with plants as well as insects and other life forms, which thus help to maintain ecosystem dynamics (Epps and Arnold 2018; Santamaria et al. 2023; Wang et al. 2025b; Wang and Zhou 2026). At the global scale, there is a growing need for standardized approaches to assess macrofungal diversity across different habitat and ecosystem types, and, crucially, to translate such biodiversity data into concrete conservation actions (Van Nuland et al. 2025; Tuula et al. 2023). However, in China, a growing number of macrofungal studies focus on the description of new species (e.g. Wu et al. 2022; Liu et al. 2024b, 2025; Wang et al. 2025a). Even if some studies conducted in China refer to the ecological distribution of macrofungi, they usually investigate species richness or the relative abundance of the funga (e.g. Deng et al. 2024), while there are fewer attempt to explore their community assembly patterns (Liu et al. 2023b). Such a situation limits the development of effective conservation strategies for macrofungi (Liu et al. 2023b). Indeed, current conservation policies in China were made mostly for protecting animals and plants, as well as the ecosystems that support them, rather than considering the unique biological characteristics and habitat requirements of macrofungi. For example, among the five national parks in China, two are established for the conservation of animals (Giant Panda National Park and Northeast China Tiger and Leopard National Park), one for forests (Hainan Tropical Rainforest National Park), and two for ecosystems (Three-River-Source National Park and Wuyishan National Park). The assessment of macrofungi in China began relatively recently, and the first Red List for macrofungi was not published until 2018 (Yao et al. 2020). Therefore, there is an urgent need to develop standardized procedures for assessing macrofungal diversity to support the identification of priority areas for fungal conservation, which would help provide scientifically grounded and practical information for policy makers on macrofungal diversity conservation.

The Hengduan Mountains, located in the southeastern Qinghai-Xizang Plateau, the western Sichuan Basin and the northwestern Yunnan-Guizhou Plateau, are a global biodiversity hotspot (Myers et al. 2000; Plateau 2014; Daru et al. 2019). The southern section of the Hengduan Mountains is listed as a priority area for biodiversity conservation in China (Xing and Ree 2017). This area possesses several climatic zones, from alpine to subtropical climates, and thus presents a distinct vertical vegetation transition, gradually from subtropical evergreen broad-leaved forests to coniferous forests, alpine shrub and alpine meadows (Sun et al. 2017). Benefitting from the highly diverse vegetation, this area also provides suitable habitats for the growth, reproduction and diversification of macrofungi (Yang 2010).

In this study, we focus on macrofungi in the southern section of the Hengduan Mountains. Based on systematic field surveys and species identification, we propose an approach to assess macrofungal diversity that includes the calculation of diversity metrics and the analysis of community assembly mechanisms. We also explore how such an approach can help identify potentially suitable priority areas for fungal conservation. Beyond the resulting macrofungal diversity data and conservation recommendations in the research region, we hope that the approach used in this study can inspire similar work in other regions of China and, ultimately, be adapted for wider use in other regions of the world and ecological contexts.

## Materials and methods

### Sampling region and strategy

This study focuses on the southern section of the Hengduan Mountains, specifically in the Daxueshan-Xiaoxiangling and Liangshan Mountains with a prominent elevation gradient ranging from 500 m to 4500 m. This region, located in the south of Sichuan Province, China (27°32'–29°32'N, 101°17'–104°23'E), is influenced by a humid subtropical monsoon climate, which brings abundant rainfall with an average annual relative humidity over 90%. Benefiting from the elevation difference and abundant rainfall, the research region is rich in vegetation types, including cold-temperate coniferous forests, temperate coniferous forests, temperate mixed conifer-broadleaf forests, warm-temperate coniferous forests, deciduous broadleaf forests, evergreen broadleaf forests, bamboo forests, evergreen broadleaf shrublands, grasslands and meadows (Sichuan Vegetation Collaboration Group 1980). To conserve these vegetation types, several nature reserves were established, including the Gongga Mountain National Nature Reserve, the Liziping National Nature Reserve, the Yele Nature Reserve, the Mabian Dafengding National Nature Reserve, the Meigu Dafengding National Nature Reserve and the Heizhugou National Nature Reserve.

**Figure 1.**
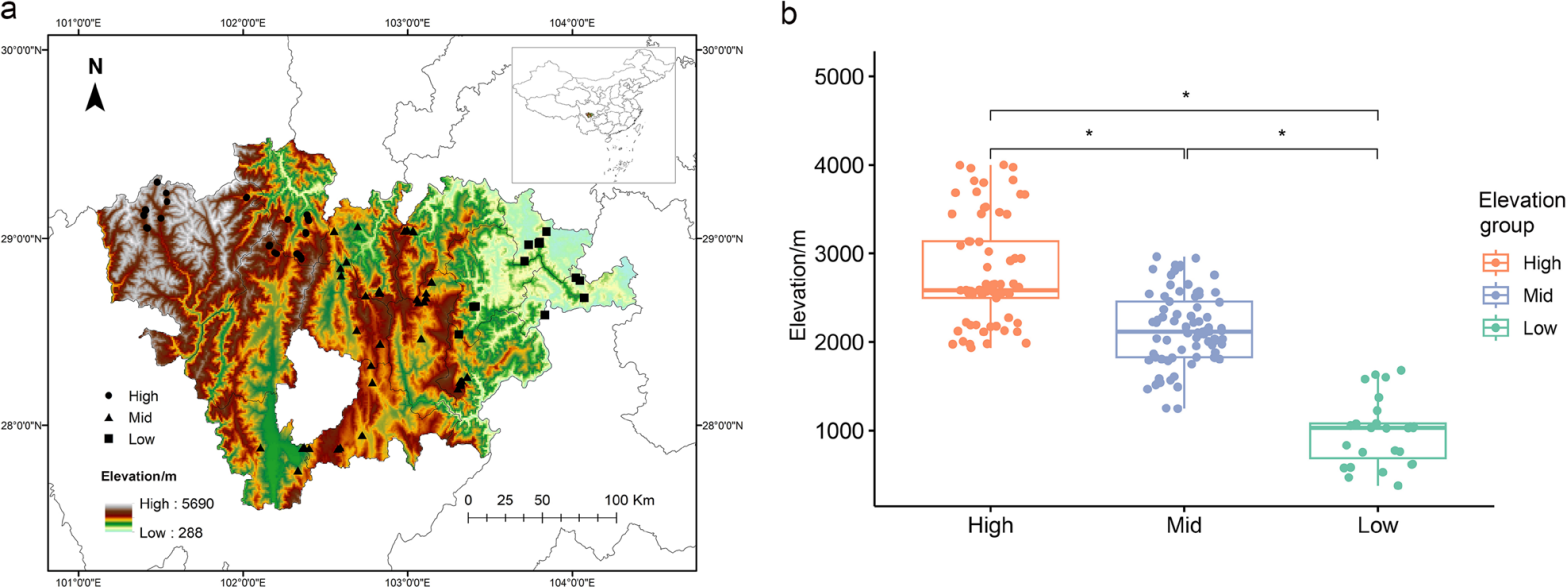
Geographical and elevational distribution of sampling sites in the southern Hengduan Mountains. **a**. Locations of the sampling sites. Circles represent sampling sites in the high elevation group, while triangles do so in the mid elevation group and squares do so in the low elevation group; **b**. Elevations of sampling sites in high, mid and low elevation groups. * indicates significant differences at the *p*-value < 0.01.

Prior to field surveys, the sampling region was divided into three groups, viz. high, mid and low elevation groups following geographic distribution from west to east (Fig. 1a). This division aligns also with major climate shifts, as well as living habitats and economic activities of local people, providing a meaningful ecological and social framework for macrofungal conservation. In July and September 2019, and August and September 2020, we performed four field trips for collecting macrofungi in the southern section of the Hengduan Mountains. The geographic locations of sampling sites were determined using a portable GPS, and then were mapped using ArcGIS 10.7 (Fig. 1a). In each sampling site (approximate 400 m^2^), collection intensity was similar, including the number of investigators (4 persons experienced in fungal inventories) and duration (5 hours). All visible macrofungi growing on various substrates, such as soil, wood, leaf litter, dung and so on, were collected. A total of 177 sampling sites, representing all typical vegetation types in the research region, were opportunistically surveyed. The high (70 sites, ranging from 1963 m to 4010 m), the mid (77 sites, from 1235 m to 2963 m), and the low elevation group (24 sites, from 377 m to 1753 m; Fig. 1b) showed significant elevation differences (*p*-value < 0.01). The collected fruiting bodies of macrofungi were dried on-site using a portable oven at 35 °C overnight. Then, the dried fruiting bodies were frozen at -80 °C for two weeks, and a total of 1771 macrofungal specimens were ultimately deposited in the Fungarium of the Institute of Microbiology, Chinese Academy of Sciences (HMAS), Beijing, China.

### Species identification

The morphological examinations of specimens followed the methods in Liu et al. (2022) and Wang et al. (2023). Briefly, macroscopic features were observed using a Leica M 125 stereomicroscope (Germany, Wetzlar) at a maximum magnification of 100×. For microscopic characteristics, specimen sections were separately prepared using cotton blue, Melzer’s reagent and 5% potassium hydroxide, and then were examined using an Olympus BX43 optical microscope (Japan, Tokyo) at a maximum magnification of 1000×.

DNA from each dried specimen was extracted using the CTAB Rapid Plant Genomic DNA Extraction Kit-DN14 (Beijing Aidlab Biotechnologies, China) following the manufacturer’s instructions. The extracted crude DNA was used as a template for PCR amplification. To amplify the ITS and nLSU regions, the primers ITS5/ITS4 (White et al. 1990) and LR0R/LR7 (Vilgalys and Hester 1990) were selected, respectively. The PCR program was as follows: for the ITS region, an initial denaturation was performed at 95 °C for 3 mins, followed by 34 cycles of denaturation at 94 °C for 40 s, annealing at 57.2 °C for 45 s and extension at 72 °C for 1 min, and a final extension at 72 °C for 10 mins; for the nLSU region, an initial denaturation was performed at 94 °C for 1 min, followed by 34 cycles of denaturation at 94 °C for 30 s, annealing at 47.2 °C for 1 min and extension at 72 °C for 1.5 mins, and a final extension at 72 °C for 10 mins. The PCR products were sequenced using the same primers at Beijing Liuhehuada Gene Technology Co. (Beijing, China). The newly generated sequences were submitted to GenBank (https://www.ncbi.nlm.nih.gov/genbank/; Suppl. material 1).

For species identification, we first used ITS and nLSU sequences of specimens as queries for BLAST searches (https://blast.ncbi.nlm.nih.gov/Blast.cgi). The species names of the best hits with a similarity greater than 98% and a coverage greater than 90% were considered reliable. In certain cases, when it was difficult to accurately identify species with BLAST searches and morphological comparisons, phylogenetic trees were constructed to assist in species identification. In general, sequences were assembled and aligned using MAFFT 7.526 (Katoh and Standley 2013) with default parameters. Then, maximum likelihood-based phylogenetic trees were constructed using raxmlGUI 2.0 (Edler et al. 2021) under the auto FC option to assess node support. Species were identified when the sequences of specimens formed well-supported clades with those of known species (bootstrap values not less than 70%).

### Community phylogenetics

We randomly selected one specimen for each species to perform phylogenetic analyses. All ITS and nLSU sequences from the selected specimens were separately aligned using MAFFT 7.526 (Katoh and Standley 2013) and manually adjusted in Aliview (Larsson 2014). Due to the presence of significant sequence differences among macrofungal species on a large taxonomic scale, conserved sites of the ITS and nLSU alignments were separately extracted using Gblocks 0.91b (Talavera and Castresana 2007), and then concatenated as a single alignment for subsequent phylogenetic analyses. Using IQ-TREE (Nguyen et al. 2015), the best-fit evolutionary model for the concatenated alignment was estimated, and the phylogenetic tree was accordingly constructed. Molecular clock dating of the phylogenetic tree topology obtained from IQ-TREE analysis was carried out using the penalized likelihood method implemented in treePL (Smith and O’Meara 2012). The following fossils were selected as phylogenetic calibration points: *Archaeomarasmius leggetti* Hibbett, D. Grimaldi & Donoghue, representing the order *Agaricales* with a minimum age of 90 million years (Hibbett et al. 1997), *Quatsinoporites cranhamii* S.Y. Sm., Currah & Stockey, representing the order *Russulales* with a minimum age of 125 million years (Smith et al. 2004), and *Paleopyrenomycites devonicus* Taylor, Hass, Kerp, M. Krings & Hanlin, representing the division of *Basidiomycota* and *Ascomycota*, with the most recent divergence time of 400 million years ago (Taylor et al. 1999). The thorough parameter was applied for sufficient iterations until convergence, and the randomcv parameter was used for cross-validation to construct a time-calibrated phylogenetic ultrametric tree. The concatenated alignment and the phylogenetic tree were submitted to TreeBASE (http://www.treebase.org; to be submitted).

Using the ultrametric tree as a template, phylogenetic diversity (PD) of macrofungi within each sampling site was determined using the PD module of Phylocom 4.2 (Webb et al. 2008). Moreover, the net relatedness index (NRI) and nearest taxon index (NTI) were selected to assess the phylogenetic structure of macrofungal community at each sampling site. NRI is a standardized measure of the average phylogenetic distance among all species pairs within a sampling site, while NTI is a standardized measure of the nearest phylogenetic distance for each species within a sampling site (Webb et al. 2002). Both indices were calculated based on 999 randomizations of species selection from the phylogenetic pool using a null model with random sampling (with replacement) implemented in the construct module of Phylocom 4.2 (Webb et al. 2008). Positive values of NRI and NTI indicate that the species composition of a sampling site is phylogenetically more clustered than expected under the null model (i.e., the community consists of more closely related species), while negative values of NRI and NTI suggest that the species composition is phylogenetically more dispersed than predicted by the null model (i.e., the community consists of more distantly related species) (Webb et al. 2002).

The null model-based method was used to quantify the relative contributions of deterministic and stochastic processes to the structure of macrofungal communities (Stegen et al. 2012). This method quantifies various ecological processes, including homogenizing selection, heterogeneous selection, homogenizing dispersal, dispersal limitation and ecological drift. The β net relatedness index (βNRI) and β nearest taxon index (βNTI) were employed to assess the relative contributions of deterministic and stochastic processes to the assembly of macrofungal communities across three elevation groups. βNRI and βNTI over 2 indicate that the phylogenetic turnover is significantly higher than expected, which is interpreted as the result of deterministic processes involving variable selection. Conversely, a value of βNRI or βNTI less than -2 suggests that phylogenetic turnover is significantly lower than expected, which is interpreted as the outcome of deterministic processes involving homogeneous selection. If |βNTI| or |βNTI| is less than 2, it indicates that the observed differences of phylogenetic composition result from stochastic processes (Tripathi et al. 2018). The Bray-Curtis-based Raup-Crick (RCbray) index was used to quantify random processes. This index represents the deviation between the observed Bray-Curtis and the null hypothesis distribution. |RCbray| > 0.95 indicates dominant homogenous diffusion (RCbray < -0.95) or diffusion limitation (RCbray > 0.95), while |RCbray| < 0.95 suggests significant deviation between the independent effects of macrofungal community turnover and drift. All analyses were conducted using the ‘NST’ and ‘iCAMP’ packages in R software (Ning et al. 2019; Ning et al. 2020).

### Identification of unique species, indicator species, and taxonomic community structure in each elevation group

Venn diagrams showing shared and unique species in the three elevation groups were generated using the Venn Diagram package in R software (Chen and Boutros 2011). Principal coordinate analysis (PCoA) was performed using the Binary Jaccard algorithm to measure the differences in species composition among the high, mid and low elevation groups (Liu et al. 2021a). To identify indicator species that can be considered as key species in each elevation group, the Dufrêne-Legendre indicator value (IndVal) method was performed using the labdsv package implemented in R software (Roberts, 2023). The statistical significance was assessed through 999 permutations (α = 0.05), and the species receiving an IndVal of more than 25% was considered to be an indicator species. In addition, to identify whether different elevation groups had significant differences in the relative abundances of different taxonomic groups, Linear Discriminant Analysis Effect Size (LEfSe) analysis (Segata et al. 2011) was performed in the R software using the microeco package (Liu et al. 2021a). The Kruskal-Wallis rank sum test (α = 0.01) was employed to screen species with significant differences among the three elevation groups, while Linear Discriminant Analysis (LDA) quantified the effect size of the differences (LDA score > 4). The results were visualized through LDA value distribution bar plots and cladograms.

### Statistical analysis and visualization

The significance of differences in species richness, PD, NRI, NTI, βNRI and βNTI among high, mid and low elevation groups was tested using the Kruskal-Wallis method in R software v 4.5.0. The results were visualized with ggplot2 in R (Wickham 2016).

## Results

### Overall composition of macrofungi

A total of 1771 macrofungal specimens were collected from 177 sampling sites in the southern section of the Hengduan Mountains during field surveys in 2019 and 2020. From these specimens, 1460 ITS and 1477 nLSU sequences were newly generated (Suppl. material 1). In association with morphological examinations, a total of 809 species, belonging to two phyla, nine classes, 28 orders, 123 families and 336 genera were identified, including one species incertae sedis at the order level, 44 species incertae sedis at the family level, and 169 species identified only to the genus level (Suppl. materials 1, 2).

**Figure 2.**
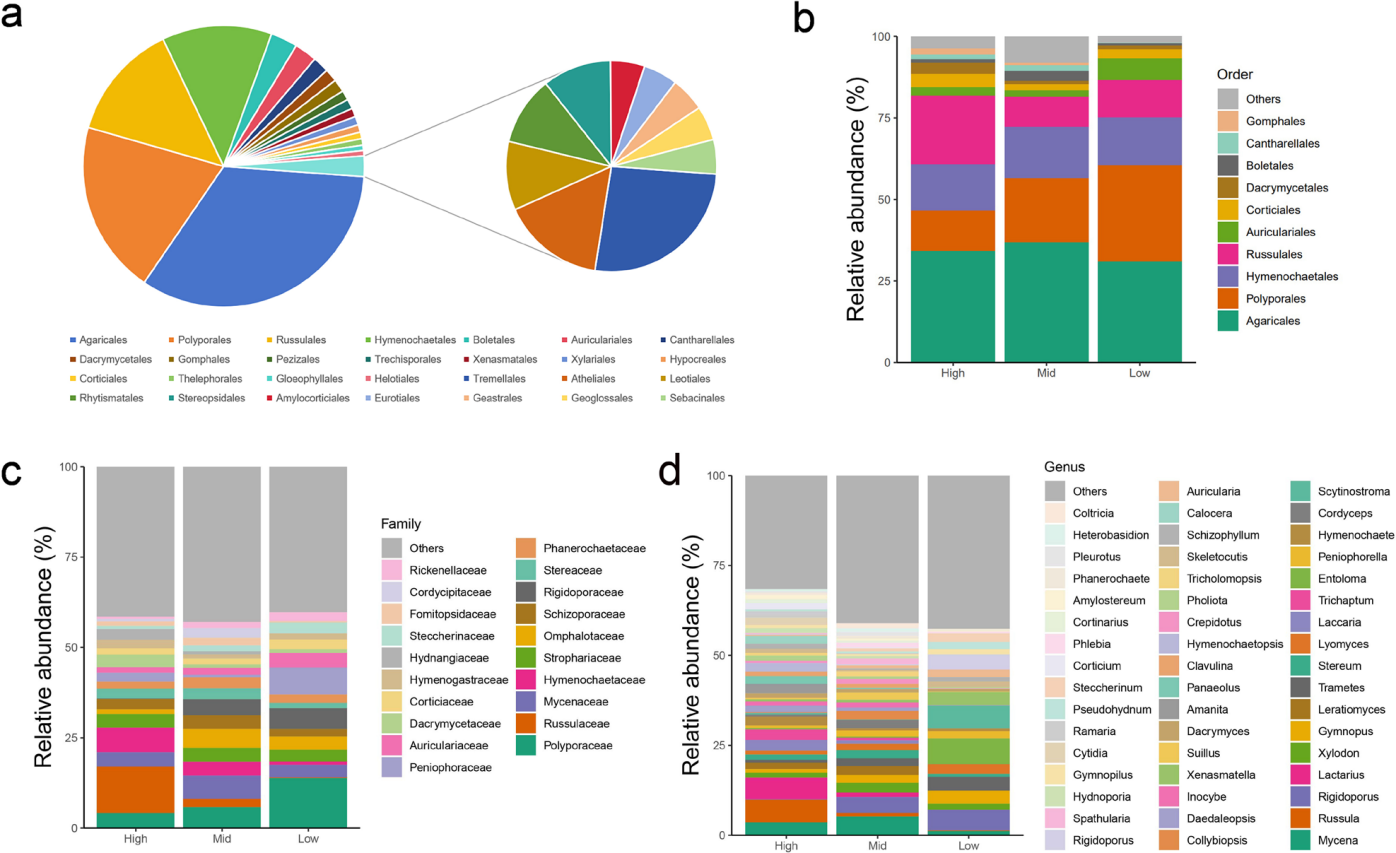
Overall composition of macrofungi in the southern section of the Hengduan Mountains; **a**. Species richness of dominant orders; **b–d**. Relative abundance of dominant orders, families and genera.

In terms of species richness, the most dominant orders are *Agaricales* (270 species), *Polyporales* (161 species), *Russulales* (109 species), *Hymenochaetales* (103 species), *Boletales* (25 species), *Auriculariales* (22 species), *Cantharellales* (15 species), *Gomphales* (12 species) and *Dacrymycetales* (12 species). The remaining 19 orders, each containing fewer than ten species, account for 10% of the total macrofungal species found in the sampling region (Fig. 2, Suppl. material 2). At the family level, 22 families consist of ten or more species of macrofungi, with *Russulaceae* (50 species) as the most speciose family (Suppl. material 2). An additional 101 families each contain fewer than ten species, accounting for 82% of total families and 32% of total species (Suppl. material 2). There are 11 dominant genera, each with ten or more species of macrofungi (Suppl. material 2). The most speciose genus is *Russula* Pers. (30 species), followed by *Xylodon* (Pers.) Gray (21 species), *Mycena* (Pers.) Roussel (19 species) and *Lactarius* Pers. (17 species). It is noteworthy that there are 60 genera each with two species and 195 genera each with one species. These 255 genera account for 76% of total genera and 39% of total species (Suppl. material 2).

When considering the specimen number of each species, viz. the relative abundance of species, we find that the dominant orders, families and genera in the whole southern section of the Hengduan Mountains as well as in each of the three elevation groups are similar to those of species richness (Fig. 2).

### The community assembly mechanism of macrofungi

A total of 700 ITS and 679 nLSU sequences were selected from all 809 identified species for phylogenetic analyses. These sequences formed a concatenated alignment matrix of 2804 characters with GTR + I + G as the optimal evolutionary model. From the generated ultrametric tree (Suppl. material 4), the species richness, PD, NRI and NTI of each sampling site were determined (Suppl. material 3).

**Figure 3.**
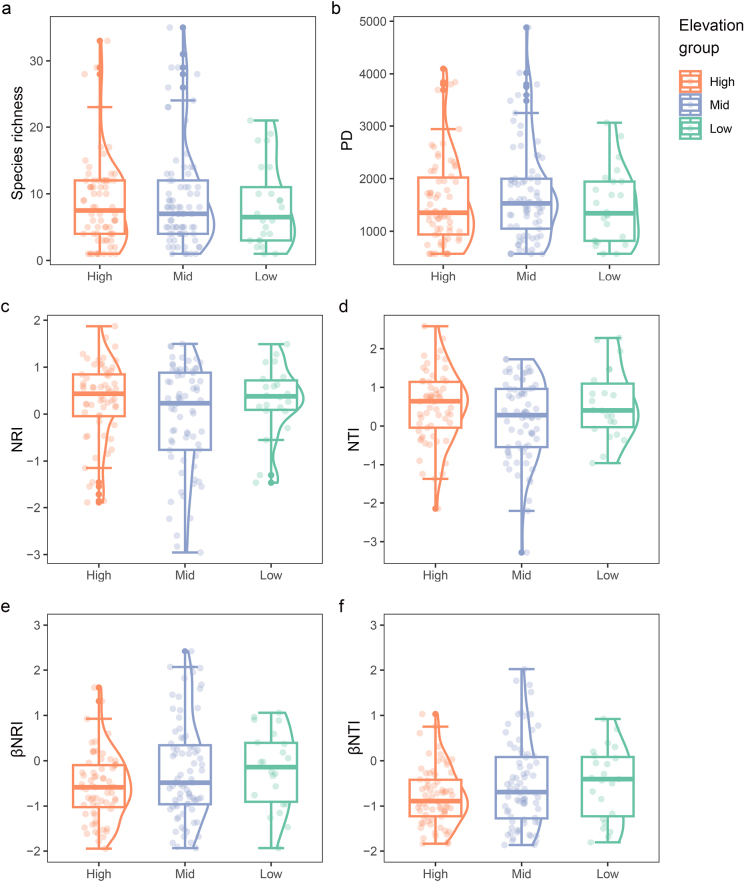
Community of macrofungi in high, mid and low elevation groups. **a**. Species richness; **b**. Phylogenetic diversity (PD); **c**. Net relatedness index (NRI); **d**. Nearest taxon index (NTI); **e**. β net relatedness index (βNRI); **f**. β nearest taxon index (βNTI).

Among the three elevation groups, species richness and PD showed no significant differences (Fig. 3a, b; *p*-value > 0.05). The NRI and NTI values of the three elevation groups were generally greater than zero, revealing the phylogenetic clustering phenomenon of macrofungal community within each group (Fig. 3c, d).

Regarding the β diversity of macrofungi in the three elevation groups, the βNRI and βNTI values of all sampling sites ranged from -2 to 2, indicating that the observed differences in phylogenetic compositions resulted from a random process (Fig. 3e, f). Furthermore, the RCbray index revealed that ecological drift contributed 93.11%, 85.79% and 80.57% to the community assembly of macrofungi in the high, mid and low elevation groups, respectively, which shows a decreasing trend of ecological drift contributions with the decrease of elevation (Table 1).

**Table 1.** The contribution of macrofungal community assembly processes to three groups in the southern section of the Hengduan Mountains.

Assembly process	High elevation	Mid elevation	Low elevation
Heterogeneous selection	1.56%	9.79%	8.47%
Homogeneous selection	2.04%	1.88%	1.63%
Dispersal limitation	0.00%	0.00%	0.00%
Homogenizing dispersal	3.29%	2.54%	9.33%
Undominated	93.11%	85.79%	80.57%

### Differential composition of macrofungi in different elevation groups

**Figure 4.**
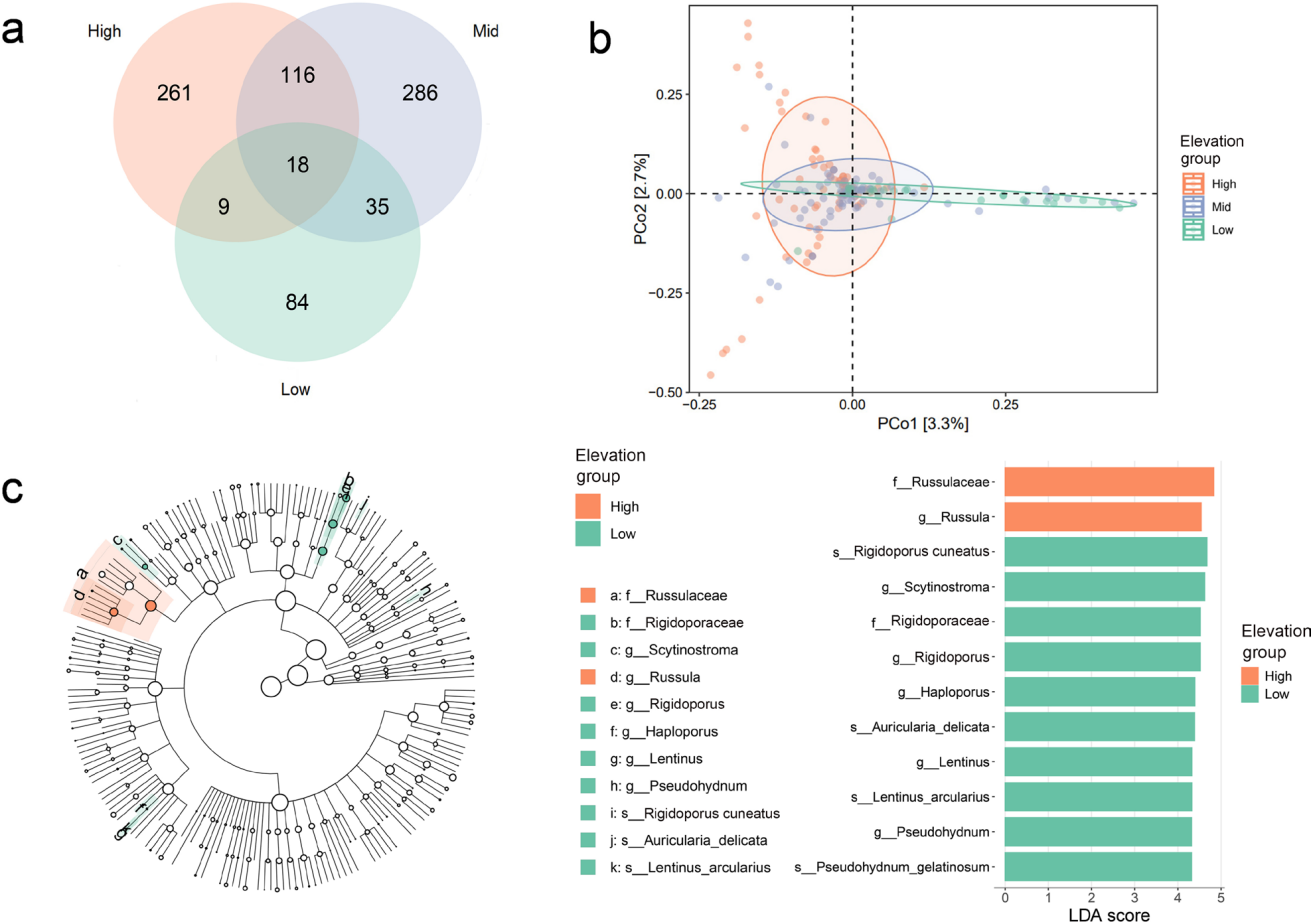
Differential composition of macrofungi in high, mid and low elevation groups. **a**. Venn diagram of species richness in different groups; **b**. Constrained PCoA plot of Bray–Curtis distances based on species richness of sampling sites in different groups. The percentage of variation indicated on each axis corresponds to the proportion of the total variance explained by the projection; **c**. Linear Discriminant Analysis Effect Size analysis indicates significantly differential composition in different groups.

The Venn diagram clearly illustrates that the mid elevation group had the most species of unique macrofungi (262 species), followed by the high elevation group (222 species), while the low elevation group had 64 unique species, which was even less than the shared macrofungi (67 species) in the three elevation groups (Fig. 4a). In addition, the high and mid elevation groups shared 126 macrofungal species, much more than either the high and low elevation groups or the mid and low elevation groups (Fig. 4a). Moreover, the PCoA result further supported that the species composition of macrofungal communities among the three elevation groups was significantly different, and the low elevation group, hosting the least number of macrofungal species, was distinct from the other two elevation groups (*p*-value < 0.05, Fig. 4b).

A total of 11 indicator species were identified following the criteria of IndVal > 0.25 and *p*-value < 0.05. Of them, *Ampulloclitocybe clavipes* (Pers.) Redhead, Lutzoni, Moncalvo & Vilgalys is exclusive in the high elevation group, while the low elevation group has *Bovista nigrescens* Pers., *Clavulina* sp. 3, *Collybia odora* (Bull.) Z.M. He & Zhu L. Yang, *Daedaleopsis confragosa* (Bolton) J. Schröt., *Haploporus* sp., *Heterobasidion orientale* Tokuda, T. Hatt. & Y.C. Dai, *Nidularia deformis* (Willd.) Fr., *Peniophorella pubera* (Fr.) P. Karst., *P. subpraetermissa* (Sheng H. Wu) K.H. Larss. and *Trichaptum fuscoviolaceum* (Ehrenb.) Ryvarden.

The LEfSe analysis revealed that the significantly differential compositions of macrofungal communities included *Russulaceae* and *Russula* in the high elevation group, and *Rigidoporaceae*, *Haploporus* Bondartsev & Singer, *Lentinus* Fr., *Pseudohydnum* P. Karst., *Rigidoporus* Murrill, *Scytinostroma* Donk, *Auricularia delicata* (Mont. ex Fr.) Henn., *Lentinus arcularius* (Batsch) Zmitr., *Pseudohydnum gelatinosum* (Scop.) P. Karst. and *Rigidoporus cuneatus* (Murrill) F. Wu, Jia J. Chen & Y.C. Dai in the low elevation group (*p*-value < 0.001; Fig. 4). These taxonomic units may be distributed in different elevation groups, but their relative abundances were particularly prominent only in specific elevation groups.

### Species diversity of macrofungi in protected and non-protected areas

**Figure 5.**
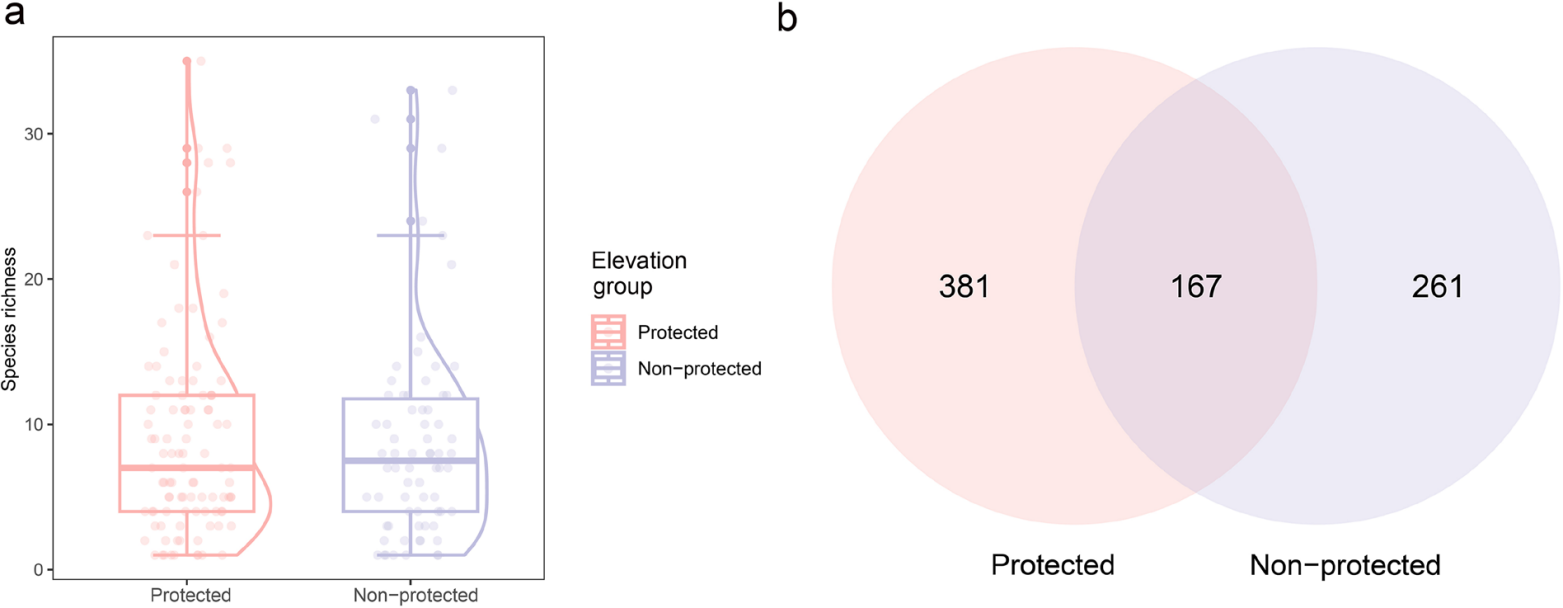
Species richness in protected and non-protected areas in the southern section of the Hengduan Mountains. **a**. Species richness of sampling sites; **b**. Unique and shared species richness.

When classifying all sampling sites based on whether they were located in a nature reserve or not, 103 sampling sites are in protected areas and 74 in non-protected areas (Suppl. material 3). The species richness in the 103 sampling sites in protected areas ranged from 1 to 35 species, while that in the 74 sampling sites of non-protected areas ranged from 1 to 33 species (Suppl. material 3). Although the number of surveyed sampling sites in non-protected areas was less than that in protected areas, no significant difference in the species richness between protected and non-protected areas was observed (Fig. 5). Moreover, there were 261 unique species to non-protected areas (Fig. 5). It is noteworthy that the new records of some endemic macrofungal species to China, such as *Hymenochaete yunnanensis* S.H. He & Hai J. Li, *Coltricia weii* Y.C. Dai and *Hyphoderma subsetigerum* Sheng H. Wu were identified in the non-protected area, viz. Xichang City, while a new record of the near threatened species *Laccaria alba* Zhu L. Yang & Lan Wang endemic to China (Wang et al. 2004; Yao et al. 2020) was found in the heavily grazed, non-protected area, viz. Mianning County.

## Discussion

Using the southern section of the Hengduan Mountains as an example, this study introduces a typical framework for assessing macrofungal diversity that can be used to identify priority areas for fungal conservation. The workflow begins with a priori stratification of the research region into meaningful units based on external criteria, such as elevation, climate, or habitat type. In our case, we pre-defined three elevation groups before any field sampling. Next, standardized field surveys are conducted within each unit to collect macrofungi, followed by integrative species identification using morphology and DNA barcoding, with voucher specimens deposited in fungaria. Subsequent biodiversity and community analyses are then performed for each unit, including species richness, phylogenetic diversity, indicator species, and community assembly mechanisms. Finally, based on these diversity metrics, such as high endemism, unique indicator species, or distinct community structure, units that most urgently require protection are identified as conservation priorities. This approach then provides scientifically grounded and practical information on where macrofungal reserves would have the highest impact.

The first thing to obtain the least biased possible view of macrofungal diversity in a given area is to conduct long-term, systematic field surveys (Halme and Kotiaho 2012; Haelewaters et al. 2021; Stallman et al. 2024). Although some areas are difficult to access due to poor transportation, four field trips over two consecutive years surveyed 177 sites across the southern section of the Hengduan Mountains. These efforts resulted in the collection of 1771 macrofungal specimens, along with detailed information for each specimen, including latitude, longitude, elevation, host plant, substrate type, etc. Compared with previous historical records of macrofungi from this region, the current collection is far more extensive, and, importantly, overcomes the spatial limitations of previous studies that focused on only a few protected areas (Ge et al. 2003; Wang et al. 2005; Wang 2012). Therefore, we have assembled the most comprehensive collection of macrofungi to date from the southern section of the Hengduan Mountains, providing a foundation for establishing a regional repository to support future diversity analyses.

To identify the 1771 macrofungal specimens, we sequenced DNA barcoding regions, viz. primarily ITS, with nLSU as a secondary marker for each specimen. Both ITS and nLSU sequences were successfully obtained from 1173 specimens, while 293 specimens yielded only ITS sequences and 305 only nLSU sequences. Combining these molecular data with morphological characteristics, 809 specimens were identified at the species level. Beyond helping identify the current specimens, this substantial sequencing effort makes an important contribution to filling gaps in global nucleotide databases (i.e., GenBank). The newly generated reference sequences, especially those from potentially new species and newly recorded local species, are a valuable resource that will improve the accuracy of future taxonomic studies and environmental DNA metabarcoding surveys in this priority area for ecological protection (Schoch et al. 2012). Indeed, of the 809 species, some were described as new in previous publications (Liu et al. 2021b, 2022, 2023a, 2024b, 2025), and some species currently identified only to the genus level will be formally described as new in the future.

Besides new species, our field surveys revealed that about half of the 809 identified species are newly recorded in the study region. From a conservation perspective, seven species are listed as threatened or near threatened on the Red List of macrofungi from China (Yao et al. 2020), including *Engleromyces sinensis* M.A. Whalley, Khalil, T.Z. Wei, Y.J. Yao & Whalley (Vulnerable, VU), *Tricholoma matsutake* (S. Ito & S. Imai) Singer (VU), *Artomyces pyxidatus* (Pers.) Jülich (Near Threatened, NT), *Cordyceps militaris* (L.) Fr. (NT), *Ganoderma applanatum* (Pers.) Pat. (NT), *Laccaria alba* (NT) and *Ophiocordyceps liangshanensis* (M. Zang, D.Q. Liu & R.Y. Hu) Hong Yu bis, Yao Wan, Y.D. Dai, Zhu L. Yang & Y.B. Wang (NT). It is noteworthy that, besides having sequences for two DNA barcoding regions, all 1771 macrofungal specimens are preserved as vouchers in HMAS, and can be morphologically re-examined and sequenced for additional gene regions at any time.

With this comprehensive and accurate species information, we performed systematic ecological analyses to explore the assembly mechanisms of macrofungal communities. Our results showed that the three elevation groups in the southern section of the Hengduan Mountains had similar macrofungal species richness or PD. The homogenized distribution pattern along the elevation gradient may reflect a broad adaptive strategy of macrofungal communities to natural environmental heterogeneity. Although species richness has long been used as a primary indicator for identifying priority areas for ecological protection, PD is another important metric that reflects the accumulated evolutionary history and the potential for future adaptation of biodiversity (Faith 1992; Winter et al. 2013). Because species richness and PD do not always align spatially, conservation strategies based solely on species richness may overlook the evolutionary significance of different lineages (Forest et al. 2007). From a macrofungal conservation perspective, it is therefore important to consider both species richness and PD, as well as lineages with unique evolutionary histories that may be particularly sensitive to threats, e.g., the Evolutionarily Distinct and Globally Endangered (EDGE) Programme (https://www.edgeofexistence.org/; Isaac et al. 2007).

Furthermore, in each of the three elevation groups, community assembly was mainly shaped by ecological habitats through a random process. While deterministic factors, such as the host identity and soil properties, are often considered to be key drivers for certain fungal groups (e.g., Jiao and Lu 2020; Xiong et al. 2021), the important role of stochasticity has also been increasingly recognized, especially in ectomycorrhizal communities (Peay et al. 2012; Tedersoo et al. 2014). Nevertheless, although the overall assembly mechanisms are similar across the three elevation groups, each group contains many unique species that are not found in other groups, and the community structures differ significantly among them. On the other hand, when comparing macrofungal species richness between protected and non-protected areas, no significant difference was found. Interestingly, the number of unique species in non-protected areas, though fewer than that in protected areas, represented 32% of all identified species across the study region. This raises the question of whether the currently established six nature reserves mentioned above truly protect the full extent of the macrofungal diversity in the southern section of the Hengduan Mountains.

From the diversity and community analyses above, the main takeaway is that the existing conservation strategy for priority ecological protection areas is likely not suitable for macrofungi in the southern section of the Hengduan Mountains. In general, it is important to protect the ecological connectivity of key habitats for macrofungal diversity across the entire priority area to avoid or at least reduce disturbances from human activities, such as freely collecting woody substrates (stumps, logs, branches and twigs) or overgrazing (Abrego et al. 2015). To achieve this, it would be beneficial to establish additional nature reserves in newly identified non-protected areas with unique macrofungal species, like Xichang. Given limited resources, conservation efforts should focus on identified indicator species, like *Ampulloclitocybe clavipes* in the high elevation group, and *Peniophorella subpraetermissa* in the low elevation group, while implementing a long-term monitoring system to track the spatiotemporal dynamics of macrofungal communities (Li et al. 2018; Stallman et al. 2024).

Although our study provides practical and feasible procedures for macrofungal diversity surveys and offers recommendations for macrofungal conservation in the southern section of the Hengduan Mountains, two issues should be considered for future assessments of macrofungal conservation in priority areas of ecological protection. The first one is to lengthen the duration of, and strengthen, the intensity of field surveys. Due to budget and time constraints, our surveys covered two years during the most suitable growing season for macrofungi (July to September). Even though we assembled the most species-rich macrofungal dataset in the study region, some species that only bear fruit in other seasons, or exist only as invisible spores or mycelia during these two years, may have been missed. Another promising avenue to fill these gaps is to employ new technologies to better capture species richness (Wang and Zhou 2024). Amplicon and metagenome sequencing can help to detect macrofungal species that would otherwise go unnoticed with traditional fruiting body-based survey methods (Taberlet et al. 2012). Previous studies have shown that neither environmental DNA sequencing nor visual fruiting body-based field surveys alone can detect all species (Frøslev et al. 2019; Cazabonne et al. 2022), and therefore combining both data sources is recommended to provide more reliable evaluations of macrofungal diversity and communities for conservation purposes (Truong et al. 2017; Copoț et al. 2024; Runnel et al. 2024). Furthermore, we note that assessing macrofungal diversity, although informing potential conservation strategies, is only a first step. How policy makers utilize such information is crucial for ensuring the effective implementation of conservation actions to protect macrofungal diversity (Lofgren and Stajich 2021; Tuula et al. 2023).

## Conclusion

We conducted systematic field surveys of macrofungi in the southern section of the Hengduan Mountains, which resulted in the collection of 1771 specimens representing 809 species. Among the high, mid and low elevation groups, there were no significant differences in species richness and PD, and community assembly was mainly shaped by ecological habitats in stochastic processes. Nevertheless, each elevation group harbored many unique species, and the community structures differed among the three elevation groups. Moreover, existing reserves in the southern section of the Hengduan Mountains do not provide effective protection for macrofungi. Therefore, we recommend establishing additional nature reserves in newly identified non-protected areas with unique macrofungal species. A long-term monitoring system can be implemented to continue to track the spatiotemporal dynamics and community assembly of macrofungal communities. Using the southern section of the Hengduan Mountains as a case study, we propose a general framework for assessing macrofungal diversity that could be used to identify priority areas for the protection of macrofungi and, ultimately, help support fungal conservation strategies.

## Additional information

### Artificial Intelligence (AI) use

The authors accept full responsibility for the content of the manuscript, including the disclosure of any use of AI.

No AI tools were used in the preparation of this manuscript.

### Adherence to national and international regulations

All the fungal strains used in this study have been legally obtained, respecting the Convention on Biological Diversity (Rio Convention).

### Author ORCIDs

J. Yu https://orcid.org/0000-0002-2851-2839

### Data availability

All of the data that support the findings of this study are available in the main text or Supplementary Information.

## Supplementary materials

Supplementary material 1 Supplementary information 1 Data type xlsx Explanation note Information on all macrofungal specimens collected in the southern section of the Hengduan Mountains. https://binary.pensoft.net/file/1677685This dataset is made available under the Open Database License (http://opendatacommons.org/licenses/odbl/1.0/). The Open Database License (ODbL) is a license agreement intended to allow users to freely share, modify, and use this Dataset while maintaining this same freedom for others, provided that the original source and author(s) are credited. Shi-Liang Liu, Dong-Mei Liu, Shan Shen, Xue-Wei Wang, Qian-Zhu Li, Jia Yu, Xing-Chun Li, Li-Wei Zhou

Supplementary material 2 Supplementary information 2 Data type xlsx Explanation note Species richness in each taxonomic unit at different taxonomic ranks. https://binary.pensoft.net/file/1677686This dataset is made available under the Open Database License (http://opendatacommons.org/licenses/odbl/1.0/). The Open Database License (ODbL) is a license agreement intended to allow users to freely share, modify, and use this Dataset while maintaining this same freedom for others, provided that the original source and author(s) are credited. Shi-Liang Liu, Dong-Mei Liu, Shan Shen, Xue-Wei Wang, Qian-Zhu Li, Jia Yu, Xing-Chun Li, Li-Wei Zhou

Supplementary material 3 Supplementary information 3 Data type xlsx Explanation note Species richness, phylogenetic diversity (PD), net relatedness index (NRI) and nearest taxon index (NTI) of all sampling sites in the southern section of the Hengduan Mountains. https://binary.pensoft.net/file/1677687This dataset is made available under the Open Database License (http://opendatacommons.org/licenses/odbl/1.0/). The Open Database License (ODbL) is a license agreement intended to allow users to freely share, modify, and use this Dataset while maintaining this same freedom for others, provided that the original source and author(s) are credited. Shi-Liang Liu, Dong-Mei Liu, Shan Shen, Xue-Wei Wang, Qian-Zhu Li, Jia Yu, Xing-Chun Li, Li-Wei Zhou

Supplementary material 4 Supplementary information 4 Data type jpg Explanation note The ultrametric tree of all 809 species identified in the southern section of the Hengduan Mountains inferred from ITS and nLSU regions. The mean divergence times are indicated at the nodes. https://binary.pensoft.net/file/1677688This dataset is made available under the Open Database License (http://opendatacommons.org/licenses/odbl/1.0/). The Open Database License (ODbL) is a license agreement intended to allow users to freely share, modify, and use this Dataset while maintaining this same freedom for others, provided that the original source and author(s) are credited. Shi-Liang Liu, Dong-Mei Liu, Shan Shen, Xue-Wei Wang, Qian-Zhu Li, Jia Yu, Xing-Chun Li, Li-Wei Zhou
